# Cutaneous responses to environmental stressors

**DOI:** 10.1111/j.1749-6632.2012.06724.x

**Published:** 2012-10-10

**Authors:** Giuseppe Valacchi, Claudia Sticozzi, Alessandra Pecorelli, Franco Cervellati, Carlo Cervellati, Emanuela Maioli

**Affiliations:** 1 Department of Evolutionary Biology, University of FerraraFerrara, Italy; 2 Kyung Hee University, Department of Food and NutritionSeoul, South Korea; 3 Department of Physiopathology, Experimental Medicine and Public Health, University of SienaSiena, Italy; 4 Department of Clinical Biochemistry, University of FerraraFerrara, Italy; 5 Department of Physiology, University of SienaSiena, Italy

**Keywords:** ozone, cigarette smoke, skin

## Abstract

Living organisms are continuously exposed to environmental pollutants. Because of its critical location, the skin is a major interface between the body and the environment and provides a biological barrier against an array of chemical and physical environmental pollutants. The skin can be defined as our first defense against the environment because of its constant exposure to oxidants, including ultraviolet (UV) radiation and other environmental pollutants such as diesel fuel exhaust, cigarette smoke (CS), halogenated hydrocarbons, heavy metals, and ozone (O_3_). The exposure to environmental pro-oxidant agents leads to the formation of reactive oxygen species (ROS) and the generation of bioactive molecules that can damage skin cells. This short review provides an overview of the effects and mechanisms of action of CS, O_3_, and UV on cutanous tissues.

## Skin and environmental pollutants

Terrestrial organisms are chronically exposed not only to natural environmental stress factors, such as ultraviolet (UV) and ozone (O_3_), but also to pollutants of anthropic origin. The skin, the largest body organ, provides the first barrier against environmental factors that physically and/or chemically can alter the body's functions. Indeed, the skin, along with the oral and respiratory tract, is the common route by which chemicals can enter the body.^1^ This protective envelope, fundamental for life on dry land and for animal evolution, consists of (a) the stratum corneum (corneocytes), which functions as a physical barrier against both percutaneous penetrations of harmful substances and excessive trans-epidermal water and salt loss; (b) a corneocytes-bound intercellular hydrophobic matrix, mainly composed of ceramides, fatty acids, and cholesterol, which forms a chemical barrier against the entry of environmental contaminants, including ambient particulate matters, as well as pathogens and allergens; and (c) an immunological barrier constituted by humoral and cellular components of the adaptive immune system, such as inflammatory cytokines and dendritic cells.

Alterations that disturb the skin barrier function in either stratum corneum lipid metabolism or protein components of the corneocytes are involved in the development of various less- or more-severe skin diseases, including erythema, edema, hyperplasia, “sunburn cell” formation, skin aging, contact dermatitis, atopic dermatitis, psoriasis, and carcinogenesis.^2^ Obviously, the protective ability of the skin is not unlimited, and problems arise when an abnormal exposure to environmental stressors exceeds the skin's normal defensive potential.

A major mechanism by which environmental insults exert a detrimental effect in the skin is through the generation of oxidative stress, which overwhelms the skin's defenses by quickly depleting the enzymatic (glutathione peroxidase, glutathione reductase, superoxide dismutase, catalase) and nonenzymatic (vitamin E, vitamin C, and glutathione) antioxidant capacity, thus leading to deleterious effects.^3^ Sun UV rays, O_3_, cigarette smoke (CS) exposure, and pollutants, in addition to the natural process of aging, contribute to the generation of free radicals and reactive oxygen species (ROS) that interact with lipid-rich plasma membrane and initiate the so-called *lipid peroxidation reaction cascade*.^4–6^ ROS also stimulate the release of pro-inflammatory mediators from a variety of skin cells. Skin inflammation, in turn, leads to skin infiltration by activated neutrophils and other phagocytic cells that generate further free radicals (both reactive oxygen and nitrogen species), thus establishing a vicious circle.^7^ Oxidative stress initiates complex biologic processes in various layers of the skin, which can result in transient or permanent genetic damage, activation of transcription factors, such as Ap1 and NF-κB,^8^ and signaling pathways, such as the ERK, JNK, and p38 MAPK pathways,^9^ involved in cell growth and differentiation and in degradation of the connective tissue of the dermis.^10^

Altered skin conditions are among the most common health problems in humans, even exceeding some of the most common pathologies such as obesity, hypertension, and cancer. More than 30% of the U.S. population has been affected by a skin disorder.^11^ And while many skin diseases are not life threatening or of sufficient societal concern and impact, they can have a significant clinical burden for individuals, affect quality of life, and account for substantial social health care costs.

The costs related to the main skin pathologies range from $157 million for cutaneous drug eruptions to $12.0 billion for skin ulcers and wounds; and the five most economically burdensome pathologies, based on direct and indirect costs, are skin ulcers and wounds, melanoma, acne, nonmelanoma skin cancer, and contact dermatitis, comprising a total of $22.8 billion in costs.^12^

Several environmental pollutants affect skin health and play a role in the pathogenesis of skin disease, among which UV, CS, and O_3_ have been shown to be the most dangerous, and thus will be discussed below.

## Skin and UV

Skin exposure to UV radiation has beneficial effects, for example in vitamin D_3_ formation, or in a curative application in combination therapy for skin diseases such as psoriasis, but it can also have many detrimental cutaneous effects, such as the development of skin malignancies. The O_3_ layer of the atmosphere efficiently absorbs solar UVB radiation. However, because of human-induced damage to the protective O_3_ layer, increasing amounts of UVB radiation reach the Earth's surface. Conversely, UVA radiation is not absorbed at all by the O_3_ layer and represents more than 95% of the solar radiation that reaches the Earth's surface. The primary mechanism by which UV radiation initiates molecular responses in human skin is via photochemical generation of ROS, which include superoxide anion (O_2_^−•^), hydrogen peroxide (H_2_O_2_), hydroxyl radical (OH^•^), and singlet oxygen (^1^O_2_). Reactive nitrogen species (RNS), such as nitric oxide (NO) and nitric dioxide, are also generated. While ROS are continuously produced in the skin by fibroblasts and keratinocytes and are involved in physiological processes, there is accumulating evidence for the damaging effects of high concentrations of ROS following UVA and UVB irradiation. In fact, although our skin possesses an array of antioxidants to either eliminate ROS or obviate their harmful effects, this capacity has limitations and can be overwhelmed.

UV radiation is one of the most important environmental factors in the development of a number of skin ailments, ranging from photo-aging to cancer, and considerable evidence over the years has shown that UV radiation triggers multiple interdependent cellular responses. UVA/B rays penetrate the skin, reach cells, and are absorbed by proteins, lipids, and nuclear and mitochondrial DNA, causing a cascade of oxidative events that can result in progressive deterioration of cellular structure and function.^13^ For example, it has been demonstrated that UV radiation, especially UVA, cause mutations of the tumor suppressor p53 gene in the basal layers of the epidermis, a region that contains keratinocyte stem cells and thus may be particularly relevant for skin carcinogenesis.^14^

Human skin is adapted for UV stress because melanocytes, which also reside in the basal layer, have the ability to produce the UV-absorbing pigment melanin, thereby protecting neighboring keratinocytes. However, following prolonged exposure to sunlight melanocytes can become targets of UV-induced carcinogenesis.^15^ In fact, excessive exposure to the UV component of sunlight is well documented to be a major risk factor in the development of both melanoma and nonmelanoma skin cancer; UV not only results in DNA damage and gene mutations but also in immune suppression, both of which are involved in carcinogenesis.^16^

In addition to direct DNA alterations, which include DNA base damage, DNA single- and double-strand breaks, and cross-linking of DNA and proteins, UVA/B-generated ROS modulate a number of signal transduction pathways (e.g., MAPKs) and transcription factors (e.g., Ap1 and NF-κB) that are important in regulating genes involved in the pathogenesis of inflammation (e.g., interleukins, iNOS, and COX-2) and in the control of the cell cycle and apoptosis (e.g., cyclin D1, Bcl2, and p53).^3^ UV radiation also affects cell membrane structures, including the activation of a number of protein kinases, transcription factors, and cell membrane receptors, to induce a variety of specific biological effects.

An important UV cellular effect in the skin is the induction of apoptosis in keratinocytes.^17^ Although UV-induced DNA damage is an important mediator of cell death, UV-induced apoptosis can be initiated both in the nucleus and at the cell membrane through direct activation of membrane-bound death receptors.^18^ UV-induced apoptosis results from at least three independent pathways: DNA damage, death receptor activation, and ROS generation, as has been previously discussed by Kulms *et al.*^19^

## Skin and cigarette smoke

Cigarette smoke (CS) is a highly complex aerosol composed of several thousand chemical substances distributed between the gas and the particulate phase. The presence of high levels of pro-oxidants, such as free radicals, in smoke is well documented, and it is estimated that gas-phase smoke contains more than 10^14^ low molecular-weight carbon- and oxygen-centered radicals per puff.^20,21^ In addition, CS contains up to 500 ppm nitric oxide (NO), which slowly undergoes oxidation to nitrogen dioxide (NO_2_).^22^ Although the radicals in gas-phase smoke have a very short life span,^21,23,24^ a wealth of evidence supports the notion that a major part of the toxicity associated with CS is related to oxidative stress caused by reactive oxidants and radical species in tobacco smoke itself or by secondary oxidative events, such as lipid peroxidation activated by smoke exposure.^25,26^

CS has been shown to affect skin health and several pathologies have been connected to CS exposure. Although it was identified more than 150 years ago,^27^ the effect of CS on skin aging was largely reported only 40 years ago (namely, increased periorbital wrinkles in smokers), and in 1985 the facial features induce by CS were defined as *smoker's face*, which describes the characteristic changes that happen to the faces of smokers, including accelerated aging facial skin with a characteristic pattern of wrinkling and sallow coloration (orange and purple color).^28^ These effects are the consequence of the activation of several mechanisms, such as induction of elastosis^29^ and upregulation of matrix metalloproteinases (MMPs-1 and -3), which degrade connective tissue (collagene, elastic fibers) and alter transforming growth factor (TGF)-β pathways.^30^ The effect of CS on cutaneous tissue is associated not only with premature skin aging and wrinkling but also with several harmful pathologies. Several epidemiologic studies have shown an association between CS and psoriasis, including a cross-sectional Norwegian study in which male smokers were found to have a significantly increased risk of developing psoriasis.^31^ Other reports have shown a dose-dependent relationship between development of psoriasis and the number of cigarettes smoked.^32^ In addition, smoking has been shown to affect the response of the patients to psoriatic treatments, thus worsening the pathology.^33^ This effect seems to be mediated by the ROS present and produced in CS; in fact, psoriatic patients have been shown to have an imbalance of oxidants and antioxidants, measured as low levels of vitamin C and glutathione (GSH), and high levels of superoxide dismutase (SOD) and malonaldehyde (MDA) in cutaneous tissues.^34^ The exact cause(s) of psoriasis is(are) not fully understood; but it is now accepted that there are three major pathological steps in its development: epidermal hyperproliferation, inflammatory infiltration of epidermis and dermis, and pathological neovasculature. It has been suggested that CS can affect all three steps. In fact, CS induces cellular proliferation from the mitogenic properties of ROS; activates immune cells as a consequence of nicotine binding to acethylcholine receptor with release of inflammatory and adhesion molecules from the immune cells;^35^ and induces pathological angiogenesis by activating the release of vascular endothelial growth factor (VEGF).^36^

A link between CS and melanoma has not been demonstrated,^37^ and CS may even have a protective role against melanoma due to its immunosuppressive properties.^38^ On the other hand, several skin cancers have been shown to be possibly related to CS exposure. For example, the development of keratoacanthoma, a variant of squamous cell carcinoma (SCC), has been clearly associated with CS exposure.^39^ But with regard to SCC^40,41^ or basal cell carcinoma (BCC),^42,43^ data are still controversial concerning exposure to CS.

## Skin and O_3_

O_3_ is another gaseous oxidant (like CS) that can induce oxidative stress in cutaneous tissues. Ozone, formed by three oxygen atoms, is present in the atmosphere and it is formed from chemical reactions between UV and O_2_. The photochemistry involved in the generation of O_3_ includes several reactions, such as photoactivation, photodecomposition, and free radical chain reaction. In fact, although O_3_ is not a radical species *per se*, its effects are mediated through free radical reactions.^44^ It is generally accepted that its noxious effects are a consequence of biomolecule oxidation, with consequent ROS generation, or via a cascade of bioactive nonradical molecules, such as aldehydes (lipid peroxidation products). The formation of these oxidation products has been shown to be prevented by antioxidant supplementation, confirming that the effect of O_3_ is mainly mediated by its ability to induce oxidative stress.

In one of the first studies on the effect of O_3_ on skin, Thiele *et al.* demonstrated that O_3_ induced significant skin depletion of vitamins E and C in concert with increased lipid peroxidation.^45^ Since O_3_ is a highly reactive molecule, it does not penetrate through cells; and in the case of cutaenous tissues, it has been shown that the first target of O_3_ is the stratum corneoum that, as mentioned before, contains a high level of unsaturated fatty acids and lipids that can be substrates for O_3_-induced peroxidation.^46,47^ In addition to increased levels of oxidative stress markers, such as lipid peroxidation, aldehydes, and protein carbonyl, and decreased antioxidant levels, such as GSH and vitamins C and E, an induction of proinflammatory markers, such as cyclooxygenase-2 (COX-2), along with increased levels of heat shock proteins (HSP-32, -70, and -27)^48^ and activation of NF-κB, were observed in skin of hairless mice exposed to 0.8 ppm of O_3_.^49^ This study was the first to show that O_3_ exposure is able to induce an active cellular response in the skin, and that O_3_ can therefore alter skin physiology. Recently, Xu *et al.* have confirmed the cutaneous toxic effect of O_3_ in humans.^50^ In this work the authors collected data from patients from urban areas of Shangai that had visited emergency rooms for skin conditions; they monitored levels of several pollutants including NO_2_, sulfur dioxide (SO_2_), particulates, and O_3_. The data, from almost 70,000 patients collected over almost two years, show a clear exposure–response relationship between increased O_3_ concentration and skin conditions such as urticaria, eczema, contact dermatitis, rash/other nonspecific eruption, and infected skin disease. Other pollutants, such as particulates, SO_2_, and NO_2_, did not show an association with skin conditions.^50^ Finally, a study by Afaq *et al.* recently showed that O_3_ effects on skin are mediated by the activation of the aryl receptor (AhR) and by the induction of the cytochrome P450 isoform CYP1, an enzyme in a detoxifying pathway usually activated in the cell by xenobiotics and carcinogens, suggesting that toxicological consequences follow the exposure of cutanous tissues to O_3_.^51^

## O_3_ and CS share similar mechanisms of action (aldehydes)

As mentioned before, O_3_ is not a radical species *per se*; instead its toxic effects are mediated through a cascade of free radical reactions. CS, on the other hand, contains more than 4,700 different chemicals, most of which, generated during the combustion process in the cigarette, are represented by ROS, RNS, and electrophilic aldehydes. Both O_3_ and CS have been shown to affect cutaneous tissues by inducing oxidative stress that leads to peroxidation.^44,52^

O_3_ cannot penetrate the skin cells but reacts instantaneously with polyunsaturated fatty acids (PUFA) to form ROS, such as H_2_O_2_, and a mixture of heterogenous LOPs, particularly unsaturated aldehydes such as 4-hydroxy-2,3-nonenal.^53,54^ In parallel, a body of evidence supports the notion that part of the oxidative stress induced by CS is due to the presence of unsaturated aldehyde species, in particular, α,β-unsaturated aldehydes present in CS (acrolein and crotonaldehyde)^52^ or formed during lipid peroxidation (4-hydroxy-2,3-nonenal and malondialdehyde),^55^ rather than to free radicals present in the CS in the gas phase. Based on the known reactivity of α,β-unsaturated aldehydes, the major modifications in proteins due to these aldehydes are likely through Michael addition, with the main nucleophilic targets being cysteine, histidine, and lysine residues.^56^ The ability of these aldehydes to modify cysteine residues has received the most attention because these residues are often involved in structural or functional protein alterations induced by oxidative events.^56^

Thus, although CS and O_3_ are chemically very different (e.g., 4,700 chemicals versus one molecule), their mechanism to induce toxicity in skin can, in part, be very similar, since they have in common several toxic messengers, such as reactive aldehydes, that induce cell toxicity.

## UV and O_3_

The skin is continuously and simultaneously exposed to several oxidative stressors that can have additive, if not synergistic, effects. While UV radiation penetrates into the epidermis (UV-B) or into the dermis (UV-A) and is known to induce the release of tissue-degrading enzymes even at suberythemal levels, O_3_ oxidizes biological systems only at the surface. Therefore, because O_3_ and UV cooperatively damage subcutaneous (SC) components they exert an additive effect in cutaneous tissues. Data have suggested that UV irradiation has been shown to compromise the skin barrier; O_3_ may enhance this phenomenon by perturbing SC lipid constituents that are known to be critical determinants of the barrier function.

Products of O_3_-induced lipid oxidation penetrate the outer skin barrier and cause effects on constituents of the deeper epidermis that can lead to activation of transcription factors, such as NF-κB, which regulates a variety of proinflammatory cytokines. On the other hand, NF-κB activation has also been implicated in the expression of collagenases by solar-simulated UV radiation and in cutaneous responses to wounding. Since O_3_ enhances UV-induced oxidation in the SC, it cannot be excluded that potentially O_3_ also enhances other UV effects, such as photo-aging.^57^ In fact, in one of the few studies that evaluated a possible additive/synergistic effect of pollutants/stressors on skin, UV and O_3_ were found to have additive effect on antioxidant depletion (vitamin E) and on lipid peroxidation levels, which could, in turn, lead to additional additive effects of these stressors.^58^

## Different responses between young and old

It has been suggested that responses to air toxicants are age related,^59^ and several recent studies have shown that, indeed, skin responses to pollutants are modulated by age. Our recent work has shown that young and old mice exposed to either O_3_ or CS have different oxidative stress and inflammatory marker responses. In fact, while both CS and O_3_ were able to induce the formation of protein carbonyls and 4-hydroxynonenal (4HNE) adducts in isolated skin, it was clear that the old mice had a higher baseline level of oxidative stress markers compared with the young mice.^60^ These results are in agreement with the free radical theory of aging by Harman, which holds that elderly individuals have higher levels of both oxidative stress and inflammatory markers (hence the term *inflammaging*).^61^ In fact, our experiments showed that the differences between young and old mice were not only in the levels of 4HNE and carbonyls adducts but also in levels of heme oxygenase-1 (HO-1) and in the expression of IL-6, IL-8, and NADPH oxidase. These data parallel two previous studies showing that old animals exposed to UV, CS, or O_3_ have an increase in the ratio of matrix metalloproteinases and tissue inhibitor of metalloproteinases (MMP/TIMP) as a consequence of increased oxidative stress,^62^ and that in a wound-healing model, elderly individuals exposed to O_3_ have a significant delay in wound-closure rates compared to young individuals.^63^

## Conflicts of interest

The authors declare no conflicts of interest.
